# Theropod courtship: large scale physical evidence of display arenas and avian-like scrape ceremony behaviour by Cretaceous dinosaurs

**DOI:** 10.1038/srep18952

**Published:** 2016-01-07

**Authors:** Martin G. Lockley, Richard T. McCrea, Lisa G. Buckley, Jong Deock Lim, Neffra A. Matthews, Brent H. Breithaupt, Karen J. Houck, Gerard D. Gierliński, Dawid Surmik, Kyung Soo Kim, Lida Xing, Dal Yong Kong, Ken Cart, Jason Martin, Glade Hadden

**Affiliations:** 1 University of Colorado Denver, Campus Box 172, Denver Colorado 80217-3364, USA; 2 Peace Region Palaeontological Research Center, Box 1540, Tumbler Ridge, British Columbia, V0C 2W0, Canada; 3 Natural Heritage Center, National Research Institute of Cultural Heritage, 927 Yudeng-ro, Seo-gu, Daejeon, 302-834, Korea; 4 National Operations Center, USDOI-Bureau of Land Management, Denver, Colorado 80225, USA; 5 Wyoming State Office, Bureau of Land Management, Cheyenne, Wyoming, 82003, USA; 6 Polish Geological Institute, ul. Rakowiecka 4, 00-975 Warszawa, Poland; 7 Department of Paleontology and Stratigraphy, Faculty of Earth Science, University of Silesia, ul. Bedzinska 60, 41-200 Sosnowiec, Poland; 8 Department of Science Education, Chinju National University of Education, Jinju, Kyungnam, 660-756, Korea; 9 School of the Earth Sciences and Resources, China University of Geosciences, Beijing 100083, China; 10 3072 Bison Ave, Grand Junction, CO, 81504, USA; 11 Downey Ct., #2, Clifton, CO, 81520, USA; 12 Bureau of Land Management, Uncompahgre Field Office, 2465 S. Townsend Ave., 81401 Montrose, Colorado, 81401, USA

## Abstract

Relationships between non-avian theropod dinosaurs and extant and fossil birds are a major focus of current paleobiological research. Despite extensive phylogenetic and morphological support, behavioural evidence is mostly ambiguous and does not usually fossilize. Thus, inferences that dinosaurs, especially theropods displayed behaviour analogous to modern birds are intriguing but speculative. Here we present extensive and geographically widespread physical evidence of substrate scraping behavior by large theropods considered as compelling evidence of “display arenas” or leks, and consistent with “nest scrape display” behaviour among many extant ground-nesting birds. Large scrapes, up to 2 m in diameter, occur abundantly at several Cretaceous sites in Colorado. They constitute a previously unknown category of large dinosaurian trace fossil, inferred to fill gaps in our understanding of early phases in the breeding cycle of theropods. The trace makers were probably lekking species that were seasonally active at large display arena sites. Such scrapes indicate stereotypical avian behaviour hitherto unknown among Cretaceous theropods, and most likely associated with terrirorial activity in the breeding season. The scrapes most probably occur near nesting colonies, as yet unknown or no longer preserved in the immediate study areas. Thus, they provide clues to paleoenvironments where such nesting sites occurred.

The close relationships between non-avian and avian theropod dinosaurs (birds) have been extensively studied and debated in recent decades^1^,^2^,^3^. Physical evidence for close relationships between these groups mostly comes from suites of osteological characters subjected to repeated cladistic analysis and refinement^1^,^2^,^3^. Feathered non-avian theropods also provide physical evidence of avian affinities^4^,^5^. However, inferences about phylogenetic relationships based on behaviour are generally more speculative. Troodontid dinosaurs buried in in avian-like sleeping postures provide rare physical evidence of stereotypical avian behaviour^6^,^7^. While other trace fossils, including nests, footprints, and bite marks also constitute behavioural evidence, they are evidently unrelated to display rituals.

While nests and eggs are clear evidence of later stages in the reproductive cycles of avian and non-avian theropods, until now it has proved impossible to demonstrate direct physical evidence of mating display behaviour in extinct non avian theropods, even though such behaviour is well-known in extant birds^8^,^9^,^10^,^11^,^12^,^13^,^14^. This means literature on dinosaur mating display behaviour has invariably been speculative^15^,^16^,^17^. For example, while one may infer that some non-avian theropods exhibited sexual dimorphism, presumably expressed in variable display behaviours, there is no physical evidence to prove such suppositions. As a result conclusive physical “evidence for sexual dimorphism in non-avian dinosaurs has been elusive”^18^ even though paleontologists infer it existed. The popular assumption that strong sexual dimorphism, in such features as crests, indicates well-developed display capacities leading to inter-or intra-sexual selection, has been challenged as simplistic by showing the importance of mutual sexual selection^19^. Nevertheless, subtle differences in oviraptosaur tail morphology are cited as evidence of sexual dimorphism, indicating tail display functions “likely employed in courtship rituals”^16^. Although potentially true, this fails to provide direct physical evidence for display behaviour. Likewise, the speculative notion that theropod courtship display behaviour led to powered avian flight^15^ has few adherents.

Here we report four sites, from a single Cretaceous rock unit in Colorado^20^ (Supplementary Information S1 and Figure S1) with extensive physical evidence of large scale scrapes, made by the left and right feet of theropod dinosaurs. We show that these scrapes likely resulted from behaviour referred to in the ornithological literature as “nest scrape display,” “nest scrape advertisement display,” “scrape ceremonies,” “pseudo nest-building,” or more generally as courtship or mating display, which is stereotypical in diverse groups of extant birds^8^,^9^,^10^,^11^,^12^,^13^,^14^. It is also reasoned that these scrapes are *not* actual nests, or the result of digging for other purposes such as searching for food, water or shelter. Thus, these scrapes represent a hitherto unrecognised category of physical trace fossil evidence ostensibly associated with early behavioural phases in the breeding cycle of non-avian theropod dinosaurs. By implication, these traces, here named *Ostenichnus bilobatus*, (“bilobed display trace”) were made in the breeding season, probably springtime. Moreover, such lek-like, “display arena”^21^,^22^ evidence links two ubiquitous classes of trace fossil: locomotion traces (repichnia) and nest sites (calichnia). In short, the display arena interpretation provides the missing link in a threefold plexus of traces that can be arranged in a coherent time sequence tied to the breeding season: trackmaking-courtship display-nesting. Although such trace-making activities do not necessarily take place at the same locations, given the stereotypical cycle of breeding behaviours among many extant avians, (congregation, display, copulation, nesting, incubation, etc.), it can be confidently inferred that abundant scrapes indicate that nest sites were established nearby. Thus, scrapes are signatures of paleogeographical significance pointing to preferred paleoenvironmental nesting sites, even as shown here where no other direct physical evidence of nesting is preserved.

## The Physical Trace Fossil Evidence

**Figure 1:**
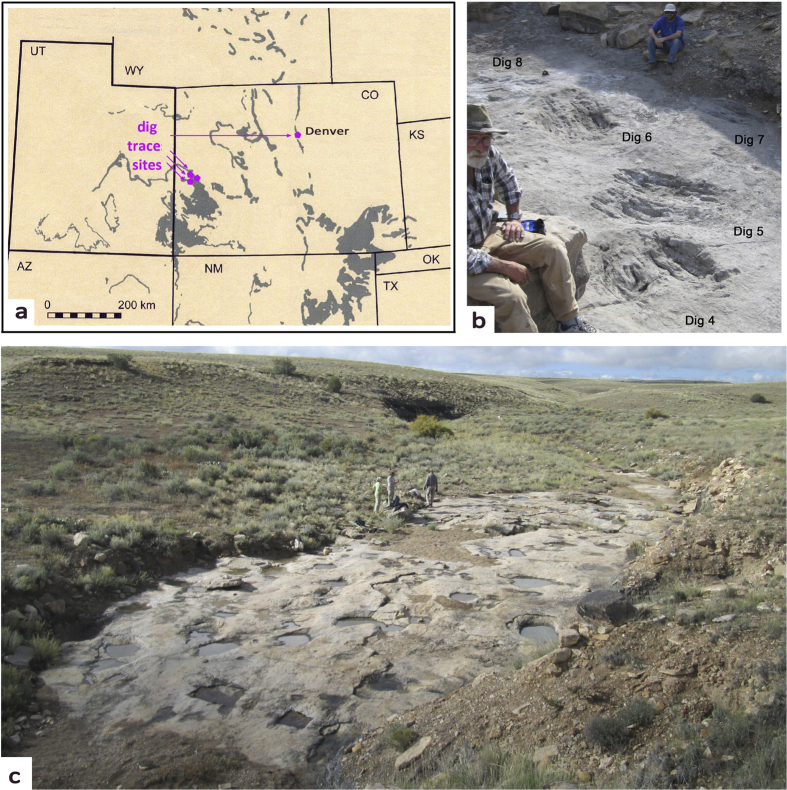
(**a**). Locality map, showing outcrops of Dakota Sandstone in western USA, extensively modified in Photoshop CS5 from part of a map by Carpenter (Supplementary Information S1) (**b**). General view of the Roubideau Creek site (photograph by senior author, M. Lockley, with coauthors KC (foreground) and JM (background) with permission. Note conspicuous scrape marks and mid-line ridge in traces 4–6, c. general view of the Club Gulch site.

**Figure 2:**
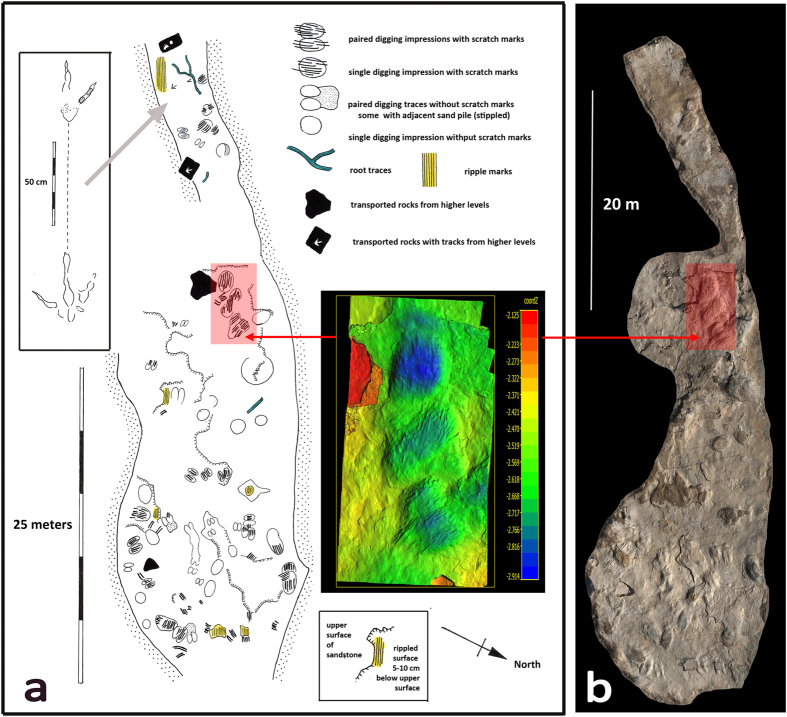
Map of Club Gulch site (**a**) prepared in Photoshop CS5 by MGL, with natural color photogrammetic image (**b**) at same scale by RTM and LGB. Coloured image (inset in a) shows three large scrapes, together covering 5 m. Digging traces are classified as paired (bilobed) or single, with or without scratch marks and adjacent sand aprons.

**Figure 3:**
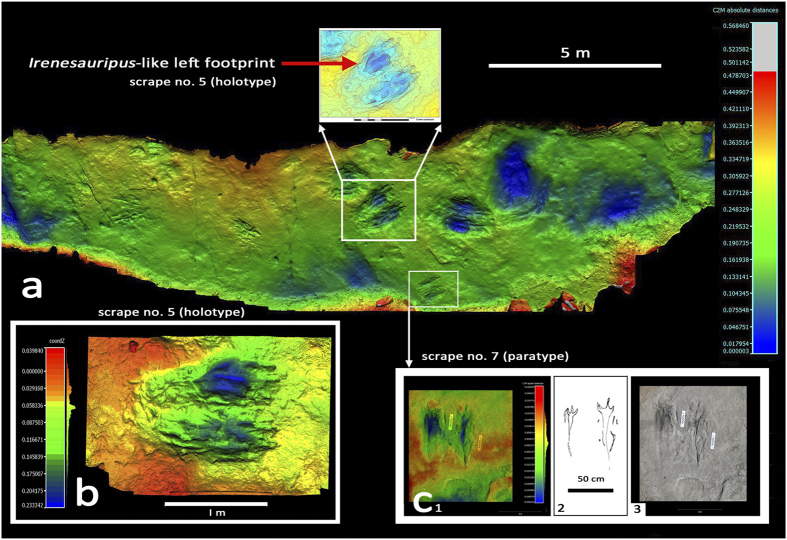
Coloured photogrammetric image of Roubideau Creek site (**a**) with location and details of holotype *Ostenichnus bilobatus* scrape (dig 5 in Fig. 1) both showing whole scrape (**b**) and contour relief detail of *Irenesauripus*-like track (A: top center). Detail of paratype scrape 7 (c _1-3_) also shows theropod track morphology.

Four sites with large nest scrape display traces have been identified in the Cretaceous (late Albian-Cenomanian), Dakota Sandstone of Colorado, three in the west, and one in the east of the state. The two largest sites are visually spectacular (Fig. 1). The Dakota Sandstone, representing a mosaic of coastal plain wetland, fluvial, lacustrine and coal swamp paleoenvironments (Supplementary Information S1), has yielded ~80 tetrapod tracksites mostly from eastern Colorado. The Dakota Group is also track-rich in the west where an additional ~40 sites have been documented^20^. Despite this abundance of footprints, display scrapes represent a previously-unrecognised, entirely new category of vertebrate trace fossil. To record this novel physical evidence all sites and representative traces have been subjected to thorough mapping and photogrammetic analyses (Figs 2 and 3: see Supplementary Information S2).

**Figure 4:**
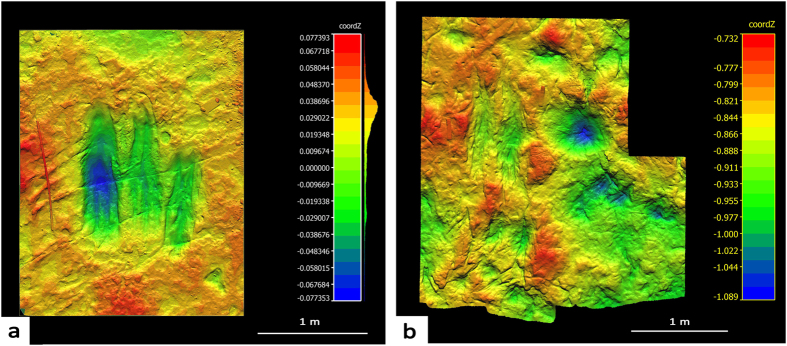
Theropod display traces from the Dakota Sandstone at Duncan Road in western Colorado (**a**) and at Dinosaur Ridge in eastern Colorado (**b**) Colorado. See Supplementary Information SI on geology and the stratigraphy of the Dakota Sandstone and Dinosaur Ridge site.

The largest site reveals ~60 scrapes on a single sandstone surface exposure up to ~50 m long and ~15 m wide (Figs 1 and 2). This area (~750 m^2^), represents a minimum estimate, for a visible surface that likely extended much further. A second site (Fig. 3) with eight well preserved scrapes occurs on a single sandstone surface ~20 × ~5 m (~100 m^2^). The size, depth and distribution of these scrapes is variable. However, most typically consist of parallel double troughs, comprised of multiple scrapes separated by a raised central ridge (see formal description). A few show complete outlines of three-toed theropod tracks, and some show thin aprons of excavated sediment aligned with the long axis of the scrapes. Two additional sites with bi- and multi-lobed *Ostenichnus bilobatus* scrapes have been found elsewhere in the Dakota Group, one in western Colorado, the other in eastern Colorado (Figs 1 and 4).

Trace fossil research recognises that traces represent “fossil behaviour”^23^ registered by living animals. General trace categories include *repichnia* for directed locomotion traces, *cubichia* for resting traces, *domichnia* for dwelling traces, *praedichnia* for predation traces and *calichnia* for breeding traces^24^. Thus, display scrapes represent a previously unrecognised type of *calichnia* trace, associated with courtship behaviour.

Some rock units, including the Dakota Sandstone^20^ are virtually devoid of tetrapod body fossil remains, yet very rich in tetrapod traces^25^,^26^. The body-fossil-poor Dakota Sandstone, has yielded tracks of avian and non-avian theropods, ornithopods, ankylosaurs, pterosaurs, crocodilians and turtles as well as diverse invertebrate traces^20^. Thus, digging traces or scrapes could potentially have been created by any of the several large tetrapods. However, the Dakota Sandstone evidence for theropod scrape-makers is compelling due to configurations indicating a bipedal trace maker with narrow acuminate claw traces. Where scrapes contain clear theropod tracks this evidence is conclusive. As no such traces have previously been reported they require the following formal systematic treatment.

## Systematics

### *Ostendichnus* ichnogen nov. Fig. 3

**Diagnosis**: large, up to 2-meter-long, bilaterally-symmetrical, bilobed to oval impressions with multiple well-defined digital scratch marks aligned parallel or sub parallel to long axis of the whole trace. Up to 10–15% as deep as long. Traces mostly with a single raised central ridge, separating left and right troughs, which may include complete or partial diagnostic tridactyl theropod tracks.

**Type material:** holotype Denver Museum of Nature and Science (DMNH) EPV.69705 latex mold and fiberglass replica of large digging trace, within which a diagnostic theropod track occurs. Paratypes DMNH EPV. 69703, EPV. 69704, EPV. 69706 and EPV. 69707 latex molds and fiberglass replicas of large digging traces (Fig. 3 and Supplementary Information 2).

**Type horizon and locality:** lower part of the Cretaceous Dakota Sandstone, Roubideau Creek, Delta County, Colorado. Information on file with DMNS, CU and BLM.

**Derivation of name:** from *ostendo* (Latin) meaning “to show”, or “to display”, and *ichnos* (Latin) meaning “a trace.”

### *Ostendichnus bilobatus* ichnosp nov., Fig. 3

**Type material:** as for ichnogenus: holotype DMNH EPV.69705 latex mold and fiberglass replica of digging trace number 5.

**Type horizon and locality:** as for ichnogenus.

**Derivation of ichnospecies name:** *bilobatus* meaning two lobes.

**Diagnosis**: as for ichnogenus.

**Description**: large, bilaterally-symmetrical, bilobed to oval impressions or scrapes 0.75 to 2.00 m long and 0.50 to 1.25 m wide; depth variable, 5 to 25 cm. Multiple well-defined digital scratch marks align with the whole trace, as does a raised medial ridge defining the long axis of the trace. Some scratch marks have sharp anterior terminations, indistinguishable from typical theropod digit traces. Together with sand crescents, where sediment was pushed back by posterior motion of digits, or thrown posteriorly as a thin apron, the left and right sides of scrapes are defined. In some scrapes complete or partial theropod tracks are recognisable components of the scrapes.

### Interpretation of Ostednichnus bilobatus

The sharply-terminated scratch marks found in association with diagnostic theropod tracks represent active theropod scraping or scratching. The most complete theropod tracks include a large *Irenesauripus*-like^27^,^28^ left theropod track on the left side of the holotype scrape (Fig. 3a) and a smaller right theropod track associated with the right side of a shallow paratype scrape (Fig. 3c). The variable size and depth of the scrapes, indicate different levels of activity and persistence by different sized theropods, which based on footprint length had hip heights between ~1.0 and ~2.0 m^29^ and full body lengths between ~2.5 and ~5.0 m. This implies either two or more different species, or co-occurrence of conspecific adults and sub-adults of quite different sizes. Elapsed time between scraping episodes cannot be estimated accurately, but was likely short given the similar, good preservation of all scrapes.

### Interpretation of nest scrape displays

Interpretation of these scrapes as evidence of mating display arenas or courtship ritual sites requires elimination of other possible digging behaviour interpretations unrelated to mating display. We can then demonstrate whether or not the behaviour, and resultant trace fossils, are consistent with behaviours of other similar or related species, in this case extant birds.

**Figure 5:**
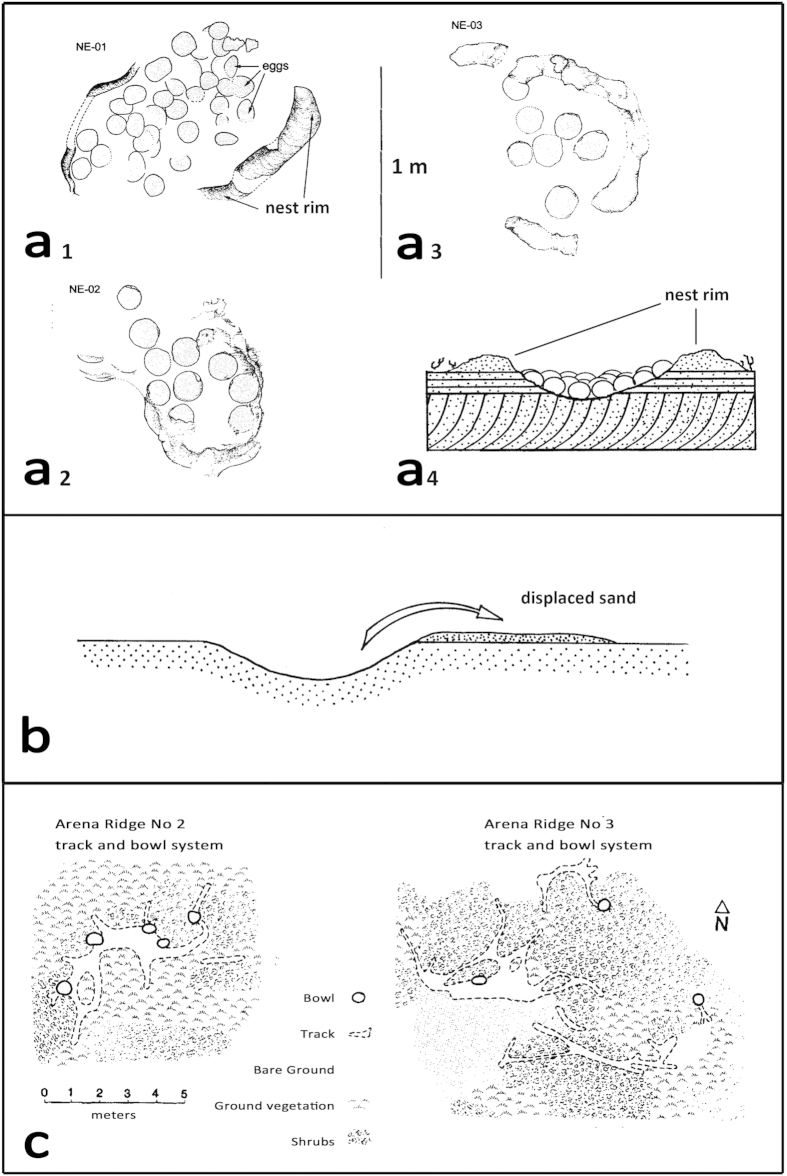
a_1_-a_3_: maps of Cretaceous sauropod nests from Argentina with cross, section (a4), after^30^. Note the nest rim in all cases. (**b**) cross section of Club Gulch dig trace with thin apron of displaced sand. Note lack of sediment rim. (**a,b**) drawn to approximately the same scale. c: map showing distribution of Kakapo nest scrape bowls, (modified after^12^).

Possible explanations for the scrapes reported from the Dakota Formation sites in Colorado, are that 1) they are actual nest sites or colonies, 2) they represent evidence of dinosaurs digging for food, water or shelter, 3) they are territory-marking scrapes, and 4) they are nuptial display arenas or scrape ceremony sites (Table 1). The nest site explanation is unconvincing because there is no evidence of eggs, eggshells, hatching remains, or the type of well-defined nest rims^30^ recorded at many nest sites (Fig. 5). Even if eggshell and hatchling remains were removed by parents or taphonomic process, the variable shapes, depths and distribution of scrapes do not conform to the typical shapes and regular-spacing configuration of nests in known dinosaur nest colonies^30^ or those of extant avians such as gannets or flamingos. It is also difficult to conceive of dinosaurs nesting, incubating and rearing young in scrapes of variable size and depth without obliterating the clear scratch marks and prominent median ridges seen in most examples. Among extant ground nesting colonial birds, such as gannets or flamingoes, nest spacing is highly regular. Nest materials are built into large mounds with preservation potential, and there are no signs of irregularly configured scrape marks. Postulating a “failed nest site” is also unconvincing because a site where theropods congregated to scrape, before moving elsewhere, leaves trace evidence essentially indistinguishable from a display arena (interpretation 4). Moreover, it requires that we assume failed nest digging attempts that are not purposeful or integral display phases in the breeding cycle. Such non-display activity would presumably waste energy, and might only be explained, tentatively, as bouts of stereotypical scrape behavior for territorial or other purposes such as testing unsuitable substrates that were later abandoned. In this regard nest scrape display is a type of territorial behavior which is better-known, documented and widespread than any scrape behavior unrelated to display.

Explanations based on digging for food or water also suffer from a lack of consistent and compelling evidence. It is known that elephants and other tetrapods dig for water^31^,^32^ or food. However, any successful attempt to dig to the level of the water table would produce pooling that would wash out scrape marks in sandy sediment. Some authors have speculated about the “scratch digging” potential of sauropods^33^,^34^. Such notions can be dismissed here because the scratch morphology fits theropods, not herbivorous sauropods. Moreover, despite ~120 known tracksites in the Dakota Sandstone^20^ the unit was deposited during the sauropod hiatus in North America^35^ when no sauropod tracks are known. Digging for prey is a plausible theropod activity supported by one diagnostic deinonychosaurid claw trace that penetrated a bioturbated zone of burrows attributed to small tetrapods, possibly mammals^36^. However, these traces bear no morphological resemblance to the large surface scrapes here described as *Ostenichnus bilobatus*, and there is also no evidence of burrows, or even buried carrion, at any of the scrape sites. Dinosaur burrows are small and very rare. A report of a ~30-cm-diameter, sub-cylindrical burrow containing an adult and two juvenile ornithopod dinosaurs^37^ could be a case of “denning,” but there is no definitive proof that a dinosaur (presumably the adult) dug the burrow in which it died. The diggers of other possible dinosaur burrows^38^ have only been inferred.

Could the scrapes be territorial markings ? Mammalian carnivores, notably cats, dig scrapes in unconsolidated sediment along game trails and mark them with urine^39^. Moreover, these traces have even been labelled “scrapes.” However, they are almost always isolated, and often along upland game trails were preservation potential is very low. Unlike ureotelic mammals, reptiles and birds are uricothelic, excreting uric acid as a final product of nitrogenous metabolism. Scent marking of territory is a mammalian trait, not known in water-conserving uricothelic reptiles and birds. This is another reason that searching for water by theropod dinosaurs is an unconvincing explanation for scrapes. In the case of the Dakota Sandstone, representing a coastal plain system with abundant evidence of saturated substrates, surface water was abundant.

Turning to the fourth hypothesis, that the scrapes represent display arenas containing evidence of ceremonial scrapes, we argue that the evidence (Table 1) consistently supports such interpretations. Ornithological literature provides many reports of scrape ceremonies and nest scrape display activity, separate from actual nest site selection and occupation. Supplementary Information 3. A compelling parallel with the Cretaceous scrapes from Colorado was reported for the Atlantic puffin (*Fratercula arctica*) in the run up to breeding, which thanks to “repeated scratching and kicking produces two parallel furrows on the floor of the burrow [with a] characteristic ridge between [that] becomes worn down and less obvious during the breeding season”^8^. The puffin is an active digger, although it often occupies and enlarges existing burrows. Unlike a large non-avian theropod it can stand more or less erect in a burrow. The traces produced by digging or scraping: i.e. two parallel furrows, with a ridge between, are strikingly similar to the theropod traces *Ostenichnus bilobatus*. The ostrich, the largest living bird, much closer in size to the Cretaceous theropod scrape-makers, produces shallow scrapes similar in size (~2–3 meters diameter) to those described here^40^ but has not been reported to engage in “nest scrape display” away from its chosen nest site, in which incubation obliterates any preservable scrape traces. These two very different birds are among the few whose scrapes have been described or illustrated, even briefly. For most of the many birds whose nest-scrape display behaviour has been documented in detail, the scrape morphology is considered an incidental behavioural bi-product, whereas the ritual ceremonies (movements) have been analysed and classified in detail^8^,^9^,^10^,^11^,^12^,^13^,^14^, often with intriguing video footage (Supplementary Information 3). At least one vertebrate ichnologist has speculated that theoretically one might find traces made by dinosaurs engaging in display behaviour, possibly even in the act of copulation^38^. Such conjectures are not new^41^,^42^ but they have been, until now, entirely speculative based only on the assumption that coition, and pre-copulation courtship, occurs in all tetrapods. However, the point is well-taken as it is the physical trace evidence, mostly ignored by modern ornithologists, that is potentially most important in paleontology^38^.

Among shorebirds (Charadriidae), nest scrape displays are reported for seven *Charadrius* species^9^ including the Wilson’s plover, *Charadrius wilsonia*^9^, banded dotterel, *Charadrius bicinctus*^10^, the piping plover (*Charadrius melodus*). Scrape displays occur from the first day a dotterel pair occupies a territory. “A male makes a scrape in sand, shuffling with his breast and kicking backwards. Shallow scrapes are often made that are never used as nests”^10^. The Knot, *Calidris canutus* engages in multiple pre-coition “nest scrape displays,” also referred to as “nest scrape advertisement displays”^11^. The importance of *Charadrius* scrapes in relation to Cretaceous theropods is that they indicate that extant, ground-nesting shorebirds make multiple scrapes, most of which are never occupied as nests. Such pre-nuptial behaviour is very energetic, expending much more energy than needed to excavate a single nest. However, it is a purposeful part of the mating ritual, not a “failed” nest construction attempt.

**Figure 6:**
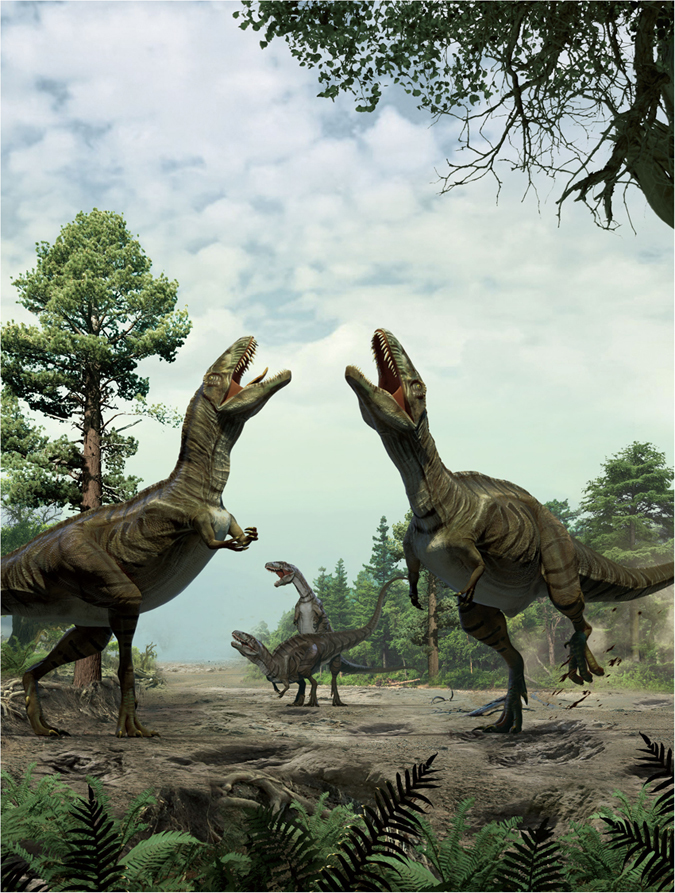
Reconstruction of theropods engaged in scrape ceremony display activity, based on trace fossil evidence from the Dakota Sandstone, Colorado. Artwork and graphics coordination by Xing Lida.

The list of substrate scraping birds is long and diverse, even if the morphology of their scrapes is only sporadically recorded (Supplementary Information 3). Studies of the Kakapo *Strigops habroptilus*, a ground-dwelling, nocturnal New Zealand parrot, demonstrate that it also digs multiple scrapes as part of its courtship rituals^12^,^43^. Studies of this species include maps showing how scrapes are irregularly distributed, from 1-5 m apart, within the male’s display area otherwise known as a lek^43^ (Fig. 5). The lek, or display arena has generated much debate among ornithologists interested in sexual selection. They define both intrasexual leks, where males display to one another, and intersexual leks where females observe male displays. The importance of mutual sexual selection has also been discussed in relation to dinosaurs and pterosaurs^19^. The ornithological literature includes maps of lek territories for many species^44^,^45^, and much importance is attached to how sexual selection drives the evolution of size, dimorphism and other characteristics in lekking species. Thus, we must consider whether the Cretaceous theropod congregation sites described here may have been analogous to certain avian lek or arena display sites^44^,^45^ as appears to be the case (Supplementary Information 3). The afore-cited studies^8^,^9^,^10^,^11^,^12^,^13^,^14^ deal with behaviour that is both territorial and sexual, being an integral part of the breeding cycle: specifically pre-nuptial courtship prior to copulation. It is intriguing to imagine the vocalizations of large theropods during courtship scrape ceremonies and copulation (Fig. 6).

## Discussion

Modern avian nest scrape displays and display arenas are created as an integral part of the breeding cycle. Avian courtship involves many stereotypical behaviours, and as such could be predicted to occur in ancestral non-avian theropods. Arguably, the Colorado scraping evidence could be the result of territorial behavior not directly related to nest scrape ceremonies. However, such an inference is problematic and unparsimonious because it infers the same stereotypical and territorial scraping behaviour used in courtship. It also infers an area of territorial dispute, with scrapes and scrape distributions that would be impossible to differentiate from a display arena. Thus, given the ubiquity of display arenas and leks among extant avians this interpretation best fits the evidence reported here. While some display arena scrape sites may represent leks of ground-dwelling birds, many leks, especially of tree-dwellers, are devoid of scrape evidence.

The Colorado evidence, points to a longevity of behaviour suggesting that theropod leks were integral to the social structure of at least some Cretaceous species. We assert that while most display behaviours (flapping, vocalization etc.,) leave no physical evidence, nest-scape displays do, and that their occurrence in the track record was even speculatively predicted^38^. Even so, a recent review of “the fossil evidence of bird behaviour”^46^ revealed little evidence of nests, and nothing about nest scrapes or display behaviour. Likewise the fossil record legacy of most behaviour is scant or ambiguous^42^. However, when made by large theropods scrapes are correspondingly large, with high preservation potential and distributions evidently extending over quite large areas. In the examples cited here the preservation potential is enhanced by sediment aggradation associated with transgressive sea-level rise^47^,^48^. This is in stark contrast to the poor preservation potential for small scrapes made in loose sand or gravel by plover-sized species, or scrapes made by ostriches in dry savannah settings. The Cretaceous evidence conforms to all predicted features of display scrapes including, variable size, depth and distribution (Table 1). Conversely, it is inconsistent with established nest sites, digging for prey or water, burrow construction or as isolated territorial scent markers. More importantly, these scrapes can be interpreted as the missing physical evidence which indicates that non-avian theropods engaged in stereotypical avian courtship and lek-like behaviours, which were previously only a matter of speculation among paleobiologists.

## Additional Information

**How to cite this article**: Lockley, M. G. *et al.* Theropod courtship: large scale physical evidence of display arenas and avian-like scrape ceremony behaviour by Cretaceous dinosaurs. *Sci. Rep.* **6**, 18952; doi: 10.1038/srep18952 (2016).

## Supplementary Material

Supplementary Information

## Figures and Tables

**Table 1:** Comparison of digging or scrape trace fossil evidence with the four working hypotheses considered here. √ indicates consistency with one or more hypotheses. x indicates lack of consistency with one or more hypotheses, ? indicates uncertain degree of consistency with one or more hypotheses.

Trace evidence	Working hypotheses (I-IV)			
Details of trace morphology	I: Nest colony hypothesis	II: Digging for food or water	III: Territorial marking	IV: Breeding / mating display
Diagnostic theropod tracks	√	√	√	√
Abundant digit and claw traces	x	x	√	√
Variable size of traces	x	√	√	√
Variable depth of traces	x	√	√	√
Variable spacing of traces	x	?	?	√
High density of traces	√	?	x	√
Frequent overlap of traces	x	?	?	√
Isolated traces	x	?	√	√
No clear tracks between traces	√	x	?	√
Few deposits of excavated sediment	?	?	?	√
No diagnostic nest rims or morphology	x	√	√	√
% of consistent evidence for hypotheses I-IV	27%	~36%	~55%	100%
