# Supplementary material for: Theropod courtship: large scale physical evidence of display arenas and avian-like scrape ceremony behaviour by Cretaceous dinosaurs
Source: Sci Rep. 2016 Jan 7;6:18952. doi: 10.1038/srep18952 (PMC4704466; doi:10.1038/srep18952)
Supplement: Supplementary Information [file srep18952-s1.pdf]

Supplementary Information for paper entitled:

Theropod courtship: large scale physical evidence of display arenas and avian-like scrape ceremony behaviour by Cretaceous dinosaurs

Martin G. Lockley[1], Richard T. McCrea[2], Lisa G. Buckley[2], Jong Deock Lim[3], Neffra A. Matthews[4], Brent H. Breithaupt[5], Karen J. Houck[1], Gerard D. Gierliński[6], Dawid Surmik[7], Kyung Soo Kim[8], Lida Xing [9], Dal Yong Kong[3], Ken Cart [10], Jason Martin [11], Glade Hadden[12]

Supplementary Information 1. GEOLOGY.

The Dakota Sandstone is a complex unit outcropping in a dozen states in the western USA, but particularly in Colorado (Fig. 1a). Locally referred to as the Dakota Formation or the Dakota Group, it is regionally subdivided into formations and members representing low-lying coastal plain deposits (main text refs [47,48]). Age determinations indicate deposition during the Albian and Cenomanian, between ~103 and 97 Million years. [1-3]

As noted below and in the methods section (**Supplementary Information S2**), the sedimentology and stratigraphy were studied in detail at two western Colorado sites: Roubideau Creek and Club Gulch (SI 1, Fig. 1). In western Colorado, the Dakota Formation is a sequence of sandstone, siltstone, and organic-rich shale with minor conglomerate, coal, and volcanic ash. It is a transgressive sequence, recording the gradual encroachment of the Western Interior Seaway. The lowermost, conglomeratic part, where the Roubideau Creek scrapes occur, is interpreted fluvial channel and swampy floodplain deposits. The middle part, where the Club Gulch scrapes occur is interpreted as crevasse splays and swampy delta plain deposits. The upper part of the formation, with thin, laterally extensive beds of sandstone separated by mud drapes, is interpreted as shoreline deposits.

The Roubideau Creek Site occurs on the upper surface of a 0.5 m unit of matrix-rich, granular, medium to coarse, sandstone occurring in the lower part of the Dakota Group **(Fig. 3, SI 1 Fig. 1)**. The sandstone comprises the upper part of a channel fill deposit approximately 1m thick and 6m wide. The surface exposure on which the scrapes were registered is about 20 meters long by 5 meters wide giving an area of about 100 m^2 and is overlain by 15 cm of coal. Eight recognizable scrapes have been identified on the sandstone surface, giving an average density of about 1 scrape per 12.5 m^2.

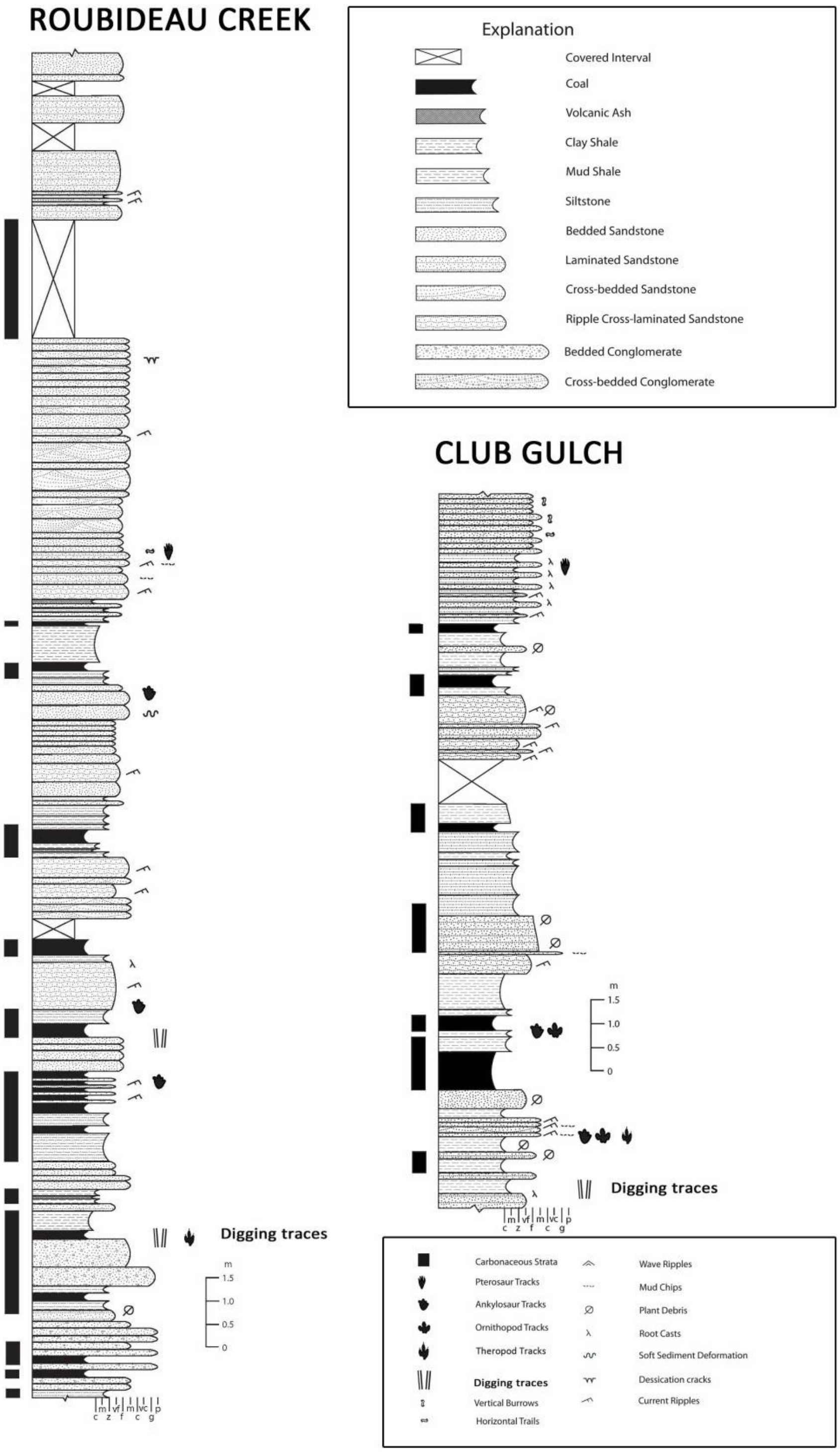

Suppl Info Fig 1. Stratigraphy of the Roubideau Creek and Club Gulch sites.

The Club Gulch site (**Figs. 1-2**) is the largest scrape site currently known. It lies in a dry wash where a bedding plane surface at the top of a thinly bedded, very fine sandstone unit is continuously exposed for a distance of about 50 meters along the drainage axis oriented down dip at about 10° WSW-ENE. The exposure is between about up to 15 meters wide giving a total area of about 50 m x 15 m = 750 m^2. The sandstone is interpreted as a crevasse splay or similar overbank deposit. The exposure reveals about 60 scrapes giving an average density of about one scrape per 12.5m^2 as shown on both the traditional and the photogrammetric maps (**Fig. 2**). These scrapes include more than 30 that have well-defined scratch marks, mostly with bilobed configurations, and about 20, more or less evenly divided between bilobed and oval depressions, which lack clear scratch marks. A few scrapes have thin, but recognizable aprons of sediment behind them. The scrape-bearing surface at Club Gulch is overlain by papery, organic rich mud shale (**SI l Fig.1**).

Most of the Club Gulch surface has been subject to various degrees of erosion, which may have been influenced by the original topography created by the scrapes. For example, at the upstream, WSW end of the site, the surface appears very smooth except for few scrapes, but further downstream there is a cluster of three large, deep scrapes with multiple scratch marks, into which runoff naturally flows. The result appears to have been a channeling of drainage into an incipient channel that has eroded the surface of the sandstone more deeply than elsewhere. In several places the uppermost layer of sandstone, which is about 5-10 cm thick, has been removed to expose small inliers revealing small scale symmetric ripple marks with a WSW-ENE ripple crest trend, on the an underlying surface (**SI 1 Fig. 1**). In most places where these ripple marks are exposed, except for the WSW end of the exposure, there is clear evidence that the inlier coincides with a scrape that has been eroded since the outcrop was exhumed. These observations on the state of preservation of the surface indicate that the number of scrapes recorded may be a minimum estimate: i.e., some may have been destroyed or rendered unrecognizable by erosion. The surface of the Club Gulch Site also reveals a few horizontal linear to gently curved, locally-branching features which we interpret as large root traces.

Two other Dakota Sandstone sites with scrapes are the Duncan Road site about 20 km east of Roubideau Creek, and Dinosaur Ridge 275 km east north east of the western slope sites. The Duncan Road sites reveals a single set of scrape marks with three sets of sub parallel groves (**Fig. 4a**) marked with multiple elongate digit traces. The three individual sets of scrapes are aligned side by side with each showing three or four distinct digit traces at the anterior end. The Dinosaur Ridge scrapes (**Fig. 4b**) have until now been regarded as enigmatic features, known to

local geologists, but not previously described in detail. They occur near the top of the Dakota Group, in deposits interpreted as those of tidal channels and mangrove swamps. Crocodylian swim tracks and fossilized log impressions are associated with the channel and swamp deposits. The scrapes are on the top of a lens-shaped body of sandstone interpreted as a tidal channel deposit. The general geology and trace fossils of the Dinosaur Ridge area have been described in many publications [3,4].

Supplementary Information 2: METHODS.

Methods used in the acquisition of data used in the description of the trace fossil sites described here fall in the following categories: 1) measuring of stratigraphic sections, 2) cleaning of sites, 3) mapping of sites by traditional means, 4) obtaining 3D photogrammetric images of sites and individual traces, and 5) molding and replication of selected trace fossils.

1) Stratigraphy and sedimentology of multiple sections were recorded in detail by one of us (KJH), in the region of the Roubideau Creek and Club Gulch sites with ichnological assistance from other authors (especially MGL) who collected track samples, molds and tracings from multiple levels. Measured stratigraphic sections were selected for optimal exposure, and where possible logged from the base of the Dakota Sandstone Formation, in its lower contact with the Cedar Mountain Formation, to its upper contact with the Mancos Shale (**SI 1**). Natural sedimentological units were measured directly with metric tape and Jacob staff. Covered units were excavated by hand with a shovel. Multiple hand samples were collected and analysed for textural and petrographic information in the lab. Where possible, bedded units were traced laterally to facilitate understanding of the potential for correlation between sections.
2) Cleaning the Roubideau Creek and Club Gulch sites prior to photography, mapping and molding work was essential because the sites lie in ephemeral, mostly dry, washes, subject to periodic deposition of alluvium following intermittent rains. The sites are not directly accessible by vehicles due to topography and Bureau of Land Management (BLM) regulations. (The Club Gulch site is within a National Conservation Area: NCA). Therefore all sites were cleaned by hand, using shovels, brushes etc., to remove alluvium (sand, silt and mud) and water accumulated after periodic rains. The Duncan

Road and Dinosaur Ridge traces occur on inclined surfaces ~20° and ~40° respectively and required little significant cleaning before photographing and mapping

3) Mapping of the Roubideau Creek and Club Gulch sites was undertaken once the sites had been thoroughly cleaned. The traditional methods involved the laying out of a grid using tape measures and Brunton compass, to plot trace fossil features onto graph paper at suitable scales (1cm to 1m, and 2cm to 1m). Individual trace fossils were recorded at a 1:1 scale by tracing features onto transparent acetate film, later reposited in the University of Colorado Museum of Natural History (UCM), along with representative molds and tracks from sites and associated stratigraphic sections. The benefits and drawbacks of this method are noted below in comparison with the photogrammetric methods employed.

4) Close range photogrammetry was employed to assist with the documentation, analyses, interpretation and graphic presentation of the digging traces at the Club Gulch, Dinosaur Ridge, Duncan Creek, and Roubideau Creek localities. The procedures followed include taking photographs in a downward orientation, perpendicular to the track surfaces (with the camera either being hand held, or mounted on a monopod and remotely triggered). Series of overlapping photographs were taken according to standard photogrammetric procedures [5,6] using a fixed focal length and aperture to ensure that intrinsic lens distortions could be characterised and accounted for by AGIsoft Photoscan Pro (v. 1.0.4) allowing for the synthesis of high-fidelity photogrammetric models. Digital surface /elevation data was exported as '.ply' and '.dem' file types into CloudCompare (v.2.5.3) to produce colour topographic maps. Global Mapper (v15.1.8) was used to generate contours.

   Specific characteristics of figured models are as follows:

   The 3D photogrammetric image of the Club Gulch track site (Fig. 2) was compiled from 1181 photographs (average elevation from subject was 2.72 m) from a Canon EOS 70D (Focal Length 18mm, resolution 5472 x 3648, pixel size 0.00417183 mm) using Agisoft Photoscan Professional (v.1.0.4) model error 0.129 pix. Color topographic maps were produced in CloudCompare (v.2.5.3).

   3D photogrammetric images of the Roubideau Creek site (Fig. 3) were compiled from 254 photographs (average elevation from subject was 1.95 m) from a Nikon D800 (Focal length 28mm,

resolution 7360 x 4912. pixel size 0.00489 mm) using Agisoft Photoscan Profesional (v.1.0.4) model error 0.149 pix. Color topographic maps were produced in CloudCompare (v.2.5.3) and topographic contours were produced in GlobalMapper (v15.1.8).

3D photogrammetric image images of the Duncan Creek site (Figure SI 3 - left) were compiled from 117 photographs (average elevation from subject was 1.07 m) from a Canon EOS 70D (Focal Length 18mm, resolution 5472 x 3648, pixel size 0.00417183 mm) using Agisoft Photoscan Professional (v.1.0.4) model error 0.141 pix. Color topographic maps were produced in CloudCompare (v.2.5.3).

3D photogrammetric image images of the Dinosaur Ridge site (Figure SI - right) were compiled from 296 photographs (average elevation from subject was 1.11 m) from a Canon EOS 70D (Focal Length 18mm, resolution 5472 x 3648, pixel size 0.00417183 mm) by Agisoft Photoscan Professional (v.1.0.4) model error 0.139 pix. Color topographic maps were produced in CloudCompare (v.2.5.3).

The advantages of the photogrammetric methods are that they produce undistorted images of the whole surface. However, these methods do not discriminate between traces (organic sedimentary structures), and inorganic sedimentary structures and weathering features. Depending on the resolution of the 3D images produced during processing of the data sets it may not be possible to discriminate features in the lab, after off -site mapping and analysis, with as much resolution as obtained by the naked eye in direct observation in the field. Traditional compass and tape methods also produce only 2D maps, but they have the advantage of allowing mappers to record only actual trace fossil features and omit non-organic features

5) Following BLM guidelines, aimed at minimizing invasive impact, molds of representative trace fossils were made after photographic images were obtained for photogrammetic analysis. At the Roubideau Creek site five large latex rubber molds of traces were made and subsequently reposited in the Denver Museum of Nature and Science as the *Ostenichnus bilobatus* holotype (DMNH EPV 69705: Fig. 3) and paratypes (DMNH EPV 69703-04 and 69706-07). After applying four coats of latex rubber and burlap fabric to all five cleaned trace fossils, the molds were covered with plastic sheets and aluminum foil in order to apply fiberglass support molds that would preserve the mold shape and not stick to the

latex. These molds were later used to make permanent "hard copy" fiberglass replicas. A single latex mold and plaster of Paris replica from the Club Gulch site is designated as UCM 207.114.

Supplementary Information 3 AVIAN ECOLOGY.

The ornithological literature is replete with information on the reproductive cycle of extant birds (avian theropods). The architecture of nests, egg color, clutch size, incubation and fledging periods are all readily studied through direct observation of the physical evidence. Likewise the study of nuptial displays, also referred to as courtship, ceremonials or mating behaviour, have been the subject of much interest and direct observation. Thus, there is no shortage of video clips showing nuptial displays involving nest scrape display (see links below). Nevertheless, none of these show the unused or abandoned scrapes left after display activity. Despite interest in how such activity might be preserved in the fossil record of Mesozoic non avian theropods (main text ref [38]) or fossil birds (main text refs.[41,42]) it has been frustratingly elusive. Courtship display behaviour, copulation (coition), nest building and pseudo-nest building are all energetic activities which imply that birds succumb to intense emotional behaviour during the breeding cycle. "Nest building…[is]…a response to an emotional drive [and]…so close is the connection between nuptial display and nest construction that Selous [7] was able to produce … facts to support his thesis that the origin of nest-building is to be found in the movement of the birds in sexual frenzy during copulation." (main text ref, [21]) Edmund Selous [7] a pioneer in avian behavioural studies first to use the term "lek" for arena display sites. He and others argued that nest-building has a "psychological basis" with a long evolutionary history, affecting diverse species. "Casting straws and scrape making figure in the … early nuptial activities of oyster catchers…[and]…black-tailed godwits,…stone curlews,…ostriches,…nightjars…and red grouse …also perform a 'scrape ceremony'… peacocks [also] scratch the ground and collect straws between bouts of displaying…"(main text ref.[21]). This indicates that these nest-building, or pseudo-nest building habits represent stereotypical avian behaviours, frequent enough to increase the abundance of trace fossils with the potential for preservation. The persistence of such stereotypical behaviours has been referred to as vestigial habits. Birds make "love and war ceremonially" and the associated tensions support "social organization." (main text ref. [21]). Birds are subject to infectious spells of excitement associated with sexual stimulation and rivalry, particularly where conspecifics, especially males, congregate at assembly grounds or leks. These assertions are supported by the extensive literature on leks and their importance in studies of sexual selection[7] (and main text refs [21,22,43-45]).

Thus, Armstrong (main ext ref .[21]) stated that "within a genus various species conform to a general, characteristic pattern of display behaviour, but show specific variations…peculiar to…one or other of the species." Moreover, "convergent evolution in display patterns may occur." Variation in body size, territory size and species specific display behaviours leads to differences in display behaviours. However, ground nesting birds that are known to scrape substrates range in size from small plovers (*Charadrius*) to ostriches (*Struthius*). Regarding the Cretaceous scrapes described here, the ostrich, the world's largest living bird, invites obvious comparison. As a ground-nesting bird close in size to theropod trackmakers from the Dakota Sandstone, it makes scrapes up to about 3 metres in diameter many of which, perhaps 80%, (main text ref. [14]) are not used as nests: i.e., they are examples of pseudo-nest building. They are also not usually considered part of a lek system as ostrich males often create them alone. Nevertheless, much lek literature dealing with ground-dwelling and/or ground nesting birds, presents maps of lek territories, which are variable in size and distribution, and differ from the spatially well-ordered nest colonies of many colonial birds and some non-avian theropods. (main text ref. [30]). Thus, we infer that the term lek could reasonably be applied to the Cretaceous scrape areas (display arenas) described here. However, we cannot presently know whether the scrapes were made intra-sexually, by rival males, inter-sexually with female observers, or as a result of mutual sexual selection activity.

The following video links show nest scrape display behavior by the species indicated:

1) Spoonbilled sandpiper. https://www.youtube.com/watch?v=1NeRF1edFT0
2) Piping Plover - https://www.youtube.com/watch?v=oyGsZflV4Jw
3) Snowy Plover scraping: https://www.youtube.com/watch?v=TPqSCMmIfyI
4) Kittlitz's Plover scrape ceremony: https://www.youtube.com/watch?v=rBJz-cj5XD8

References

1) Carpenter, K. Where the sea meets the land: the unresolved Dakota Problem in Utah. *In* Maclean, J. S., Biek, R. F., & Huntoon, J. E. (eds) *Geology of Utah's far south*. *Utah Geol. Assoc. Pub*.**43**, 257-372. (2014).

2) Barclay, R., S., Rioux, M., Meyer, L. B.,. Bowring, S. A., Johnson, K. AS., Miller, I. M.. High precision U-Pb zircon geochronology for Cenomanian Dakota Formation floras in Utah. *Cret. Res.,* **52**, 213-217 (2015).

3) Lockley M. G., and Marshall, C. *A field guide to the Dinosaur Ridge Area*. [40] Friends of Dinosaur Ridge, Morrison, Colorado, (2014).

4) Weimer, R. J., Land, C. B. Jr., MacKenzie, D. B., Harms, J. C., & Walker, T.R. Environments of Sandstone deposition, Colorado Front Range. *Mountain Geol*., **9**, 239-267. (1972).

5) Matthews, N. A., T. A. Noble, and B. H. Breithaupt. The application of photogrammetry, remote sensing and geographic information systems (GIS) to fossil resource management; pp. 119-131 in S. G. Lucas, J. A. Spielmann, P. Hester, J. P. Kenworthy, and V. L. Santucci, (eds.), Fossils from Federal Lands. *New Mexico Mus. Nat. Hist. Sci. Bull*. **34**, :119 131 (2006).

6) Petti, F.M., Avanzini, M., Belvedere, M., de Gasperi, M. de, Ferretti, P., Girardi, S., Remondino, F., & Tomasoni, R. Digital 3D modelling of dinosaur footprints by photogrammetry and laser scanning techniques: integrated approach at the Coste dell' Anglone tracksite (Lower Jurassic, southern Alps, northern Italy). *Studi Trent. Sci. Nat., Acta Geol*., 83, : 303-315 (2008).

7) Selous, E. Observations tending to throw light on the question of sexual selection in birds. *The zoologist*. ser 4, vol. 11, no. 788, p. 60-65, & no 791, p. 161-182 (1907).
